# Explainable Artificial Intelligence in the Early Diagnosis of Gastrointestinal Disease

**DOI:** 10.3390/diagnostics12112740

**Published:** 2022-11-09

**Authors:** Kwang-Sig Lee, Eun Sun Kim

**Affiliations:** 1 AI Center, Korea University Anam Hospital, Seoul 02841, Korea; 2 Department of Gastroenterology, Korea University Anam Hospital, Seoul 02841, Korea

**Keywords:** gastrointestinal disease, early diagnosis, artificial intelligence

## Abstract

This study reviews the recent progress of explainable artificial intelligence for the early diagnosis of gastrointestinal disease (GID). The source of data was eight original studies in PubMed. The search terms were “gastrointestinal” (title) together with “random forest” or ”explainable artificial intelligence” (abstract). The eligibility criteria were the dependent variable of GID or a strongly associated disease, the intervention(s) of artificial intelligence, the outcome(s) of accuracy and/or the area under the receiver operating characteristic curve (AUC), the outcome(s) of variable importance and/or the Shapley additive explanations (SHAP), a publication year of 2020 or later, and the publication language of English. The ranges of performance measures were reported to be 0.70–0.98 for accuracy, 0.04–0.25 for sensitivity, and 0.54–0.94 for the AUC. The following factors were discovered to be top-10 predictors of gastrointestinal bleeding in the intensive care unit: mean arterial pressure (max), bicarbonate (min), creatinine (max), PMN, heart rate (mean), Glasgow Coma Scale, age, respiratory rate (mean), prothrombin time (max) and aminotransferase aspartate (max). In a similar vein, the following variables were found to be top-10 predictors for the intake of almond, avocado, broccoli, walnut, whole-grain barley, and/or whole-grain oat: *Roseburia* undefined, *Lachnospira* spp., *Oscillibacter* undefined, *Subdoligranulum* spp., *Streptococcus salivarius* subsp. *thermophiles*, *Parabacteroides distasonis*, *Roseburia* spp., *Anaerostipes* spp., Lachnospiraceae *ND3007* group undefined, and *Ruminiclostridium* spp. Explainable artificial intelligence provides an effective, non-invasive decision support system for the early diagnosis of GID.

## 1. Introduction

### 1.1. Gastrointestinal Disease

Gastrointestinal disease (GID) is a major cause of disease burden in the world [1,2,3,4,5,6]. GID is defined as the disease of the gastrointestinal tract, e.g., the esophagus, liver, stomach, small and large intestines, gallbladder, and pancreas. Common GIDs are gastroesophageal reflux disease (GERD), cancer, irritable bowel syndrome, lactose intolerance, and hiatal hernia. Their common symptoms are bleeding, bloating, constipation, diarrhea, heartburn, nausea, pain, and vomiting [1]. GID is reported to contribute to 8 million deaths across the globe every year [2] and USD 120 billion of total expenditure in the United States as of 2018 [3]. Likewise, its disability-adjusted life years (1730 per 100,000, 5.9%) ranked 8th among 21 disease groups in Korea for the year 2015 [4], whereas its medical cost amounted to USD 4 billion or 13% of all medical costs in the country for the year 2007 [5]. GID has a variety of causes including: (1) bad health behavior, e.g., low-fiber diet, insufficient exercise, disrupted routine, high-dairy diet, excessive stress; (2) unhealthy bowel habits; (3) excessive anti-diarrheal/antacid medication; and (4) pregnancy [6].

There are two types of GID, functional and structural. In the case of functional GID, the gastrointestinal tract looks normal but reveals motility problems in medical examination. Its common examples include bloating, constipation, diarrhea, gas, GERD, irritable bowel syndrome, nausea, and poisoning. In the case of structural GID, the gastrointestinal tract has the issues of an abnormal outlook and motility at the same time. Colorectal polyps, colorectal cancers, diverticular disease, hemorrhoids, inflammatory bowel disease, stenosis, and strictures belong to the category of structural GID. GID can be prevented based on sound health behaviors, healthy bowel habits, and regular health screening such as regular colonoscopies from the age of 45. For instance, a majority of colorectal cancers develop when colorectal polyps, non-cancerous growths of colorectal tissues, begin to invade their surrounding tissues. Most of these colorectal polyps can be removed with no pain based on colonoscopy, whereas more advanced colorectal cancers require more complex surgical operations [1,6].

### 1.2. Explainable Artificial Intelligence

Recently, the notions of artificial intelligence and machine learning have garnered global attention. The definition of artificial intelligence is “the capability of a machine to imitate intelligent human behavior” (the Merriam–Webster dictionary). As a division of artificial intelligence, machine learning is denoted as “extracting knowledge from large amounts of data” [7]. The artificial/deep neural network, the decision tree, the naïve Bayesian predictor, the random forest, and the support vector machine are popular machine learning approaches (See [7] for a detailed explanation of these approaches). Specifically, a random forest is a group of decision trees which make majority votes on the dependent variable (“bootstrap aggregation”). Let us take a random forest with 1000 decision trees as an example. Let us assume that the original data include 10,000 participants. Then, the training and test of this random forest takes two steps. First, new data with 10,000 participants are created based on random sampling with the replacement, and a decision tree is created based on these new data. Here, some participants in the original data would be excluded from the new data, and these leftovers are called out-of-bag data. This process is repeated 1000 times, i.e., 1000 new data are created, 1000 decision trees are created, and 1000 out-of-bag data are created. Second, the 1000 decision trees make predictions on the dependent variable of every participant in the out-of-bag data, their majority vote is taken as their final prediction on this participant, and the out-of-bag error is calculated as the proportion of wrong votes on all participants in the out-of-bag data [7]. An artificial neural network is a group of neurons (information units) that are networked based on weights. It normally has one input layer, one, two, or three intermediate layers, and one output layer. A deep neural network is an artificial neural network with a large number of intermediate layers, e.g., 5, 10, or even 1000 [8].

**Figure 1:**
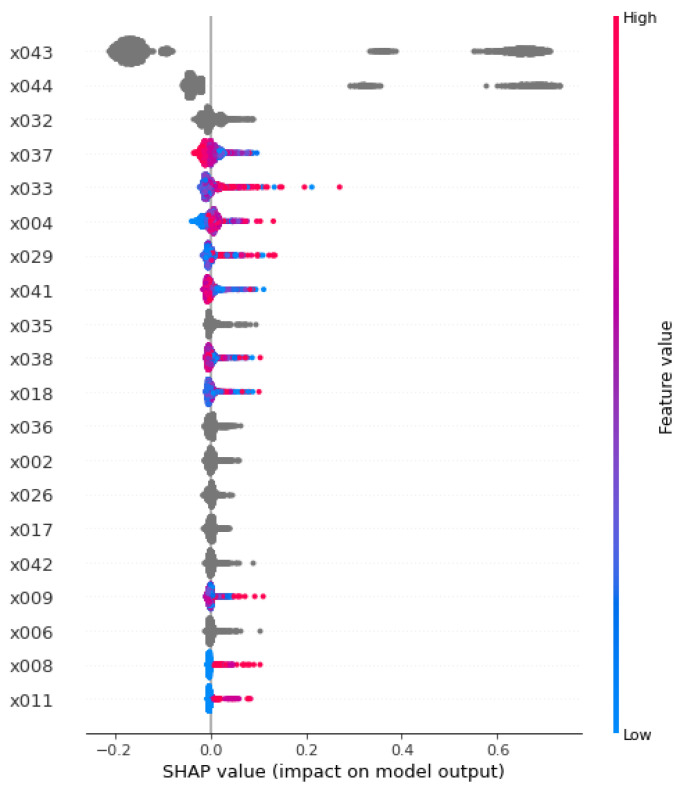
SHAP summary plot. The SHAP value of a predictor for a participant measures the difference between what machine learning predicts for the probability of GERD with and without the predictor. For example, in this hypothetical figure, the SHAP values of diabetes (x033) for GERD have the range of (−0.05, 0.30). Here, some participants have SHAP values as low as −0.05, and other participants have SHAP values as high as 0.30. The inclusion of a predictor (diabetes) into machine learning will decrease or increase the probability of the dependent variable (GERD) by the range of −0.05 and 0.30. In other words, there exists a positive association between diabetes and GERD in general.

Conventional research covers a limited range of predictors for the early diagnosis of disease, using logistic regression with an unrealistic assumption of *ceteris paribus*, i.e., “all the other variables staying constant”. For this reason, emerging literature employs artificial intelligence for the early diagnosis of disease, e.g., arrhythmia [8], birth outcome [9,10], cancer [11,12], comorbidity [13], depression [14], liver transplantation [15], menopause [16,17], and temporomandibular disease [18,19]. It is free from unrealistic assumptions of “all the other variables staying constant”. It delivers the importance values and rankings of predictors for the early diagnosis of the dependent variable. Moreover, the notion of explainable artificial intelligence is enjoying immense popularity now. Explainable artificial intelligence can be defined as “artificial intelligence to identify major predictors of the dependent variable”, and there are four approaches of explainable artificial intelligence at this point, i.e., random forest impurity importance, random forest permutation importance [20,21], machine learning accuracy importance, and Shapley additive explanations (SHAP) [15,22,23,24,25,26,27,28,29,30,31,32]. Random forest impurity importance calculates the node impurity decrease from the creation of a branch on a certain predictor. It is a sum over all trees in a random forest with the range of 0 and the number of all trees. Random forest permutation importance measures the overall accuracy decrease from the permutation of data on the predictor. It is an average over all trees in the random forest with a value of 0 to 1 [20,21]. Machine learning accuracy importance (an extension of random forest permutation importance) calculates the accuracy decrease from the exclusion of data on the predictor. The SHAP value of a predictor for a participant measures the difference between what machine learning predicts for the probability of GID with and without the predictor [15,22,23,24,25,26,27,28,29,30,31,32]. For example, let us assume in a hypothetical figure (Figure 1) that the SHAP values of diabetes (x033) for GERD have the range of (−0.05, 0.30). Here, some participants have SHAP values as low as −0.05, and other participants have SHAP values as high as 0.30. The inclusion of a predictor (diabetes) into machine learning will decrease or increase the probability of the dependent variable (GERD) by the range of −0.05 and 0.30. In other words, there exists a positive association between diabetes and GERD in general. Random forest impurity importance and random forest permutation importance had been the only explainable artificial intelligence methods before machine learning accuracy importance, and the SHAP was introduced as their extension or alternative very recently.

In practice, experts in artificial intelligence use random forest impurity importance, random forest permutation importance, or machine learning accuracy importance to derive the rankings and values of all predictors for the prediction of the dependent variable. Then, they employ the SHAP plots to evaluate the directions of associations between the predictors and the dependent variable. Linear or logistic regression used to play this role before the SHAP approach took it over. This is because the SHAP approach has a notable strength compared to linear or logistic regression: the former considers all realistic scenarios, unlike the latter. Let us assume that there are three predictors of GERD, i.e., age, diabetes, and (calcium channel blocker) medication. As defined above, the SHAP value of diabetes for GERD for a particular participant is the difference between what machine learning predicts for the probability of GERD with and without diabetes for the participant. Here, the SHAP value for the participant is the average of the following four scenarios for the participant: (1) age excluded, medication excluded; (2) age included, medication excluded; (3) age excluded, medication included; and (4) age included, medication included. In other words, the SHAP value combines the results of all possible sub-group analyses, which are ignored in linear or logistic regression with an unrealistic assumption of *ceteris paribus*, i.e., “all the other variables staying constant”. In this context, the purpose of this study is to review the recent progress of explainable artificial intelligence for the early diagnosis of GID.

## 2. Methods

**Figure 2:**
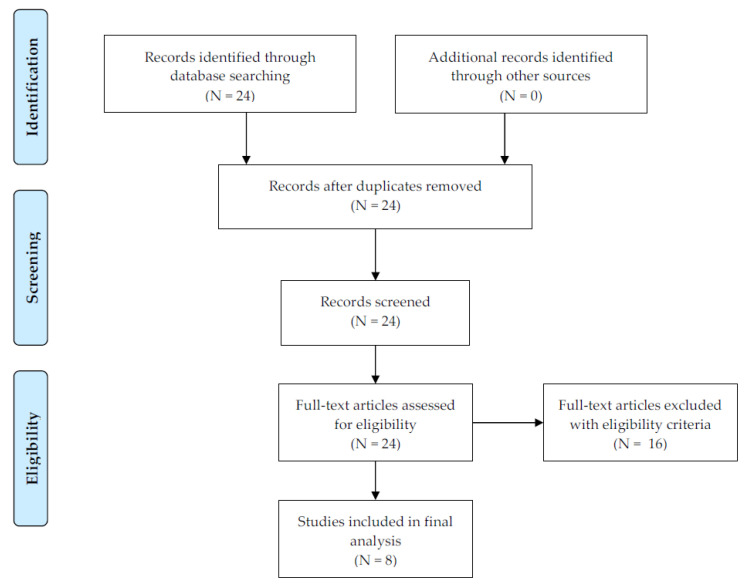
Flow diagram.

Figure 2 shows the flow diagram of this study. Eight original studies were selected for review out of twenty-four original studies in PubMed with the search terms “gastrointestinal” (title) together with “random forest” or ”explainable artificial intelligence” (abstract). The inclusion criteria of this review were: (1) the intervention(s) of the artificial/deep neural network, the decision tree, the naïve Bayesian predictor, the random forest, and/or the support vector machine; (2) the outcome(s) of accuracy and/or the area under the receiver operating characteristic curve for the early diagnosis of GID or a strongly associated disease; (3) the outcome(s) of variable importance and/or the SHAP for the early diagnosis of GID or a strongly associated disease; (4) a publication year of 2020 or later; and (5) the publication language of English. The following summary measures were adopted: artificial intelligence methods, sample size, data type, performance measures, and important predictors. Accuracy denotes the proportion of correct predictions over all observations. The area under the receiver operating characteristic curve (AUC) represents the area under the plot of the true positive rate (sensitivity) against the false positive rate (1-specificity) at various threshold settings.

## 3. Results

### 3.1. Summary

The summary of the review for the eight original studies [33,34,35,36,37,38,39,40] is presented in Table 1. The table includes five summary measures such as artificial intelligence methods, sample size, data type, performance measures, and important predictors (independent variables). The ranges of performance measures were reported to be 0.70–0.98 for accuracy, 0.04–0.25 for sensitivity, and 0.54–0.94 for the AUC. The following determinants were discovered to be top-10 predictors of gastrointestinal bleeding in the intensive care unit: mean arterial pressure (max), bicarbonate (min), creatinine (max), PMN, heart rate (mean), Glasgow Coma Scale, age, respiratory rate (mean), prothrombin time (max), and aminotransferase aspartate (max). In a similar vein, the following factors were found to be top-10 predictors for the intake of almond, avocado, broccoli, walnut, whole-grain barley, and/or whole-grain oat: *Roseburia* undefined, *Lachnospira* spp., *Oscillibacter* undefined, *Subdoligranulum* spp., *Streptococcus salivarius* subsp. *thermophiles*, *Parabacteroides distasonis*, *Roseburia* spp., *Anaerostipes* spp., Lachnospiraceae *ND3007* group undefined, and *Ruminiclostridium* spp. The most important predictors for the prediction of early intestinal resection with Crohn’s disease were clinical variables of age and disease behavior as well as the single nucleotide polymorphisms of rs28785174, rs60532570, rs13056955, and rs7660164. However, artificial intelligence is a data-driven approach, and more research is needed for more general conclusions.

### 3.2. Numeric Data

This section summarizes original studies with numeric data regarding explainable artificial intelligence for the early diagnosis of GID or a strongly associated disease. A recent study [33] used single-center data and random forest permutation importance for the prediction of preterm birth, which has a strong association with GERD. Data on 36 demographic, socioeconomic, and clinical determinants came from Anam Hospital in Seoul, Korea, with 731 obstetric patients during January 1995—August 2018. In terms of accuracy, the random forest (0.8681) was similar with the logistic regression (0.8736). Based on random forest permutation importance, the major predictors of preterm birth were age (0.1211), education (0.0332), upper gastrointestinal tract symptom (0.0274), GERD (0.0242), Helicobacter pylori (0.0151), and region (0.0139). Likewise, a follow-up study [38] employed population data and random forest impurity importance to confirm these findings. Retrospective cohort data on 29 demographic, socioeconomic, and clinical determinants came from Korea National Health Insurance Service claims data for all women who were aged 25–40 years and gave birth for the first time as a singleton pregnancy during 2015–2017 (405,586 women). According to random forest impurity importance, the main predictors of preterm birth during 2015–2017 were socioeconomic status in 2014 (240.28), age in 2014 (221.13), GERD for the years 2012 (42.24), 2014 (38.86), 2010 (37.76), 2013 (36.64), 2007 (35.01), and 2009 (34.39), region in 2014 (34.36), and GERD for the year 2006 (31.98). These studies conclude that preterm birth has a stronger association with GERD than it does with periodontitis, and it would be vital to promote active counseling for general GERD symptoms (neglected by pregnant women).

A recent study [34] used multi-center data and the SHAP for the prediction of mortality from gastrointestinal bleeding in the intensive care unit. The source of the data on 34 demographic and clinical factors was 5691 patients of gastrointestinal bleeding registered in the Electronic Intensive Care Unit Collaborative Research Database. The XGBoost outperformed the APACHE IVa for prediction: specificity 0.27 vs. 0.04 at 1.00 sensitivity; AUC 0.85 vs. 0.80. Based on the SHAP, the major predictors of mortality from gastrointestinal bleeding in the intensive care unit were mean arterial pressure (max), bicarbonate (min), creatinine (max), PMN, heart rate (mean), Glasgow Coma Scale, age, respiratory rate (mean), prothrombin time (max), aminotransferase aspartate (max), albumin (min), oxygen saturation (mean), white blood cell, AlkPhos (max), platelet (min), lactate (max), intubation, bilirubin (max), international normalized ratio (max), vasopressor, glucose (max), blood urea nitrogen (max), PTT (max), hemoglobin (min), and potassium. In conclusion, explainable artificial intelligence provides an effective, non-invasive decision support system for the prediction of high-risk gastrointestinal bleeding in the intensive care unit.

Two recent studies [39,40] highlight the effectiveness of explainable artificial intelligence in investigating strong associations of gastrointestinal factors with COVID-19 hospitalization or infection. The first study [39] employed single-center data and random forest permutation importance for the prediction of COVID-19 hospitalization based on gastrointestinal factors. Data on 19 demographic and clinical variables came from the University Hospital in Martin, Slovakia, with 710 participants in the COVID-19 test during February 2021–May 2021. The AUC range of the random forest was (0.76, 0.80). Based on random forest permutation importance, the major predictors of COVID-19 hospitalization were aspartate transaminase (0.1451), diabetes mellitus (0.0248), chronic liver disease (0.0169), alanine transaminase (0.0110), diarrhea (0.0068), age (0.0139), and bloating (0.0011). In a similar vein, the second study [40] utilized single-center data and random forest permutation importance for the prediction of gastrointestinal sequelae months after COVID-19 infection. The source of data on 23 demographic and clinical variables was the University Hospital in Martin, Slovakia, with 590 participants in the COVID-19 test during February 2021–October 2021. The AUC of the random forest was 0.68. According to random forest permutation importance, the main predictors of gastrointestinal sequelae months were acute diarrhea (0.066) and antibiotics administration (0.058).

### 3.3. Genomic and Radiomic Data

This section summarizes original studies with genomic and radiomic data regarding explainable artificial intelligence for the early diagnosis of GID or a strongly associated disease. A recent study [35] used existing literature and random forest permutation importance for the prediction of intake for almond, avocado, broccoli, walnut, whole-grain barley, and whole-grain oat. The data on 4375 amplicon sequence variants came from five randomized control trials with 340 observations on microbiota composition. The accuracy and AUC of the random forest were 0.70 and 0.92, respectively. Based on random forest permutation importance, the top 10 predictors for the intake of almond, avocado, broccoli, walnut, whole-grain barley, and/or whole-grain oat were *Roseburia* undefined (0.097), *Lachnospira* spp. (0.043), *Oscillibacter* undefined (0.039), *Subdoligranulum* spp. (0.039), *Streptococcus salivarius* subsp. *thermophiles* (0.039), *Parabacteroides distasonis* (0.032), *Roseburia* spp. (0.026), *Anaerostipes* spp. (0.023), Lachnospiraceae *ND3007* group undefined (0.022), and *Ruminiclostridium* spp. (0.022).

A recent study [36] employed multi-center data and the SHAP for the prediction of early intestinal resection with Crohn’s disease. The source of the data on seven demographic/clinical factors and 102 single nucleotide polymorphisms was the IMPACT Study with 337 Crohn’s disease patients during May 2017–May 2020. The AUC range of the CatBoost was (0.81, 0.84). Based on the SHAP, the major predictors of early intestinal resection with Crohn’s disease were the clinical variables of age and disease behavior as well as the single nucleotide polymorphisms of rs28785174, rs60532570, rs13056955, and rs7660164. Another study [37] utilized single-center data and random forest permutation importance for the prediction of pneumatosis. The source of data on four radiomic factors was the radiological reports of 71 pneumatosis patients between 2012 and 2019. The accuracy range of the random forest was (0.78, 0.94). According to random forest permutation importance, the main predictors of pneumatosis were dissecting gas in the bowel wall (0.19), intramural gas beyond a gas–fluid/faecal level (0.15), and a circumferential gas pattern (0.12). These studies conclude that explainable artificial intelligence, together with genomic or radiomic data, also provides an effective, non-invasive decision support system for the prediction of GID or a strongly associated disease.

## 4. Discussion

Previous studies on the early diagnosis of GID based on explainable artificial intelligence had some limitations. Firstly, existing literature was characterized by single-center data with small sample sizes. Using multi-center or population data (e.g., national health insurance claims data) will further the horizon of research in this direction. Secondly, the AUC of some studies (0.68) might not be optimal as a diagnostic test yet. Thirdly, the four approaches of explainable artificial intelligence at this point (i.e., random forest impurity importance, random forest permutation importance, machine learning accuracy importance, and SHAP) can lead to different results in certain circumstances. Random forest impurity importance can vary depending on how variables are categorized, whereas random forest permutation importance is relatively free from this possible variation [21]. This would explain why only one of the eight original studies reviewed here used random forest impurity importance. It can be noted, however, that the random forest has a unique strength of incorporating sequential information and that this strength is much more apparent with impurity importance than with permutation importance. In this context, a comprehensive comparison for the four approaches of explainable artificial intelligence would be a great contribution for this line of research. Fourthly, the eight original studies reviewed above were selected with the search terms “gastrointestinal” (title) together with “random forest” or ”explainable artificial intelligence” (abstract). These terms would be quite specific or broad. Employing a greater variety of search terms and comparing their results would make a great contribution to this line of research. Fifthly, this review did not consider other types of explainable artificial intelligence including local interpretable model-agnostic explanations (LIME) [41].

Indeed, some suggestions for this line of research are presented here. Firstly, combining different types of explainable artificial intelligence for different types of GID data would break new ground and bring more profound clinical insights. An increasing number of research endeavors combine image, genetic, and numeric artificial intelligence for disease diagnosis, prognosis, prevention, and management (wide and deep learning). This strand of research involves the extensive employment of multi-input multi-out models with Tensorflow or Keras. For example, one recent study [42] developed a glaucoma prediction system based on convolutional neural networks extracting key image features from multiple video inputs and recurrent neural networks predicting glaucoma outcomes from the trajectory of the key image features over time. In the convolutional neural network, feature detectors slide across input data, and their detections of certain features (their operations of “convolution”) predict the status of a cell as normal vs. GID. In the recurrent neural network, the current output is determined, in a “recurrent” pattern, by the current input and the previous hidden state (here, the previous hidden state is the memory of all the past inputs) [7,8]. Little literature is available, and more examination is needed regarding the combination of different types of explainable artificial intelligence for different types of GID data.

Secondly, little research has been conducted and more examination is needed on explainable artificial intelligence for reinforcement learning. Reinforcement learning is a branch of machine learning in which (1) the environment presents a series of rewards, (2) an agent takes a series of actions to maximize the cumulative reward in response, and (3) the environment moves to the next period with given transition probabilities [43]. In fact, it has been reinforcement learning that has brought the notion of artificial intelligence to worldwide popularity since the publication of a seminal article on Alpha-Go in 2016. Two revolutionary ideas behind reinforcement learning were that artificial intelligence (e.g., Alpha-Go) starts like a human player, i.e., takes a series of actions and maximizes the cumulative reward (chance of victory) from the limited information available in limited periods only, and that it moves far beyond the best human player ever based on the sheer power of big data covering all human players to date. In other words, it is reinforcement learning (or temporal difference learning in a professional language) that epitomizes the salient characteristics of artificial intelligence as “being similar with but superior to human intelligence” [43]. Reinforcement learning has gained immense popularity in finance given that it does not require unrealistic assumptions but does register superb performance compared to conventional statistical models [44]. This success has been replicated in healthcare, covering treatment recommendation, diagnosis automation, resource allocation, and other domains of service in chronic disease and critical care alike from both structured data and unstructured information [45]. However, little literature has been available, and more investigation is needed on explainable reinforcement learning. A recent review reports that there have been a few studies on this issue, and these studies have relied on simplified models with easy interpretation but insufficient performance and little consideration of the psychological and social factors behind optimization processes [46].

In summary, this study reviewed the recent progress of explainable artificial intelligence for the early diagnosis of GID. The ranges of performance measures were 0.70–0.98 for accuracy, 0.04–0.25 for sensitivity, and 0.54–0.94 for the AUC. The following determinants were top-10 predictors of gastrointestinal bleeding in the intensive care unit: mean arterial pressure (max), bicarbonate (min), creatinine (max), PMN, heart rate (mean), Glasgow Coma Scale, age, respiratory rate (mean), prothrombin time (max), and aminotransferase aspartate (max). The following factors were top-10 predictors for the intake of almond, avocado, broccoli, walnut, whole-grain barley, and/or whole-grain oat: *Roseburia* undefined, *Lachnospira* spp., *Oscillibacter* undefined, *Subdoligranulum* spp., *Streptococcus salivarius* subsp. *thermophiles*, *Parabacteroides distasonis*, *Roseburia* spp., *Anaerostipes* spp., Lachnospiraceae *ND3007* group undefined, and *Ruminiclostridium* spp. Likewise, most important predictors for the prediction of early intestinal resection with Crohn’s disease were the clinical variables of age and disease behavior as well as the single nucleotide polymorphisms of rs28785174, rs60532570, rs13056955, and rs7660164. In conclusion, explainable artificial intelligence provides an effective, non-invasive decision support system for the early diagnosis of GID.

## Figures and Tables

**Table 1:** Summary of review.

ID	Method	Sample Size	Data Type	Performance	Important Predictor
[33]	ANN DT LR * NB RF * SVM	731	Numeric	Accuracy 0.79–0.87 AUC 0.54–0.76	RFVI for the prediction of preterm birth, which has a strong association with GERD: Age, education, upper gastrointestinal tract symptom, Helicobacter pylori, region
[34]	APACHE XGB *	5691	Numeric	Sensitivity 1.00 Specificity 0.04–0.27 AUC 0.80–0.85	SHAP for the prediction of mortality from gastrointestinal bleeding in the intensive care unit: mean arterial pressure (max), bicarbonate (min), creatinine (max), PMN, heart rate (mean), Glasgow Coma Scale, age, respiratory rate (mean), prothrombin time (max), aminotransferase aspartate (max), albumin (min), oxygen saturation (mean), white blood cell, AlkPhos (max), platelet (min), lactate (max), intubation, bilirubin (max), international normalized ratio (max), vasopressor, glucose (max), blood urea nitrogen (max), PTT (max), hemoglobin (min), potassium
[35]	RF *	340	Genomic	Accuracy 0.70 AUC 0.92	RFVI for the prediction of food intake (almond, avocado, broccoli, walnut, whole-grain barley, whole-grain oat): *Roseburia* undefined, *Lachnospira* spp., *Oscillibacter* undefined, *Subdoligranulum* spp., *Streptococcus salivarius* subsp. *thermophiles*, *Parabacteroides distasonis*, *Roseburia* spp., *Anaerostipes* spp., Lachnospiraceae *ND3007* group undefined, *Ruminiclostridium* spp.
[36]	CB *	337	Genomic	AUC 0.81–0.84	SHAP for the prediction of early intestinal resection with Crohn’s disease: age, disease behavior (clinical predictors), rs28785174, rs60532570, rs13056955, rs7660164 (single nucleotide polymorphisms)
[37]	RF *	71	Radiomic	Accuracy 0.78–0.94	RFVI for the prediction of pneumatosis: dissecting gas in the bowel wall, intramural gas beyond a gas-fluid/fecal level, a circumferential gas pattern
[38]	ANN * LR * RF *	405,586	Numeric	Accuracy 0.93–0.98	RFVI for the prediction of preterm birth, which has a strong association with GERD: socioeconomic status, age, region (city)
[39]	RF *	710	Numeric	AUC 0.76–0.80	RFVI for the prediction of COVID-19 hospitalization based on gastrointestinal factors: aspartate transaminase, diabetes mellitus, chronic liver disease, alanine transaminase, diarrhea, age, bloating
[40]	RF *	590	Numeric	AUC 0.68	RFVI for the prediction of gastrointestinal sequelae months after COVID-19 infection: acute diarrhea, antibiotics administration

ANN—Artificial Neural Network, AUC—Area under the Receiver Operating Characteristic Curve, CB—CatBoost, DT—Decision Tree, LR—Logistic Regression, NB—Naïve Bayes, RF—Random Forest, RFVI—Random Forest Variable Importance, SHAP—Shapley Additive Explanations, SVM—Support Vector Machine, XGB—XGBoost, * Method with the best performance.
